# The Extremely Enhanced Photocurrent Response in Topological Insulator Nanosheets with High Conductance

**DOI:** 10.1186/s11671-018-2758-0

**Published:** 2018-11-21

**Authors:** Shiu-Ming Huang, Lin-Jie Lin, You-Jhih Yan, Shih-Hsun Yu, Mitch M. C. Chou, Ho-Feng Hsieh, Chin-Jung Ho, Ruei-San Chen

**Affiliations:** 1 Department of Physics, National Sun Yat-Sen University, Kaohsiung, 80424 Taiwan; 2 Department of Materials and Optoelectronic Science, National Sun Yat-Sen University, Kaohsiung, 80424 Taiwan; 3 Center of Crystal Research, National Sun Yat-sen University, Kaohsiung, 80424 Taiwan; 4 Graduate Institute of Applied Science and Technology, National Taiwan University of Science and Technology, Taipei, 10607 Taiwan

**Keywords:** Nanosheets, Photoresponse, Topological insulator

## Abstract

The photocurrent was performed in topological insulator nanosheets with different conductances. The higher photocurrent is observed in the nanosheet with higher conductance. The responsivity is proportional to the nanosheet conductance over two orders. The responsivity is independent of the light power intensity in vacuum, but responsivity drastically decreases at low power intensity in air. The ratio of the responsivity in air to that in vacuum is negatively proportional to the the inverse of the light power intensity. These behaviors are understood as the statistical photocurrent in a system with blocked molecules. The time constant decreases as the thickness increases. A longer time constant is observed in lower atmosphere pressure.

## Introduction

It is an ongoing task to look for materials with higher photocurrent response. The short light penetration depth in solid-state materials leads to that the photocurrent response is dominated by surface carriers. A material with higher abundant surface carrier is a better candidate as a photodetector. For a long time, materials with high surface-to-volume ratios, such as nanowires, were widely studied [1–6]. Accompanied with the wide photodetection bandwidth, low-dimensional materials with linear E-K dispersion, such as graphene, [7, 8] graphene-based heterostructures, [1–4], two-dimensional transitional metal dichalcogenides (TMDs), and topological materials, have attracted wide attention [9–16].

The recent reports reveal that the reported photocurrent response varies in wide ranges [17–22]. One intuitively ascribes these distributions to different material growth and experimental conditions. Most of the reports focus their attention on the material component adjustment. The potential intrinsic mechanisms on these distributions are less investigated and discussed. Clarifying the intrinsic mechanism might help one improve potential defect and greatly optimize the performance. It is believed that the sample quality should be a critical factor dominating the photocurrent response [17–22]. In addition to the crystal structure and component analysis, are there any other simple physical methods to determine the sample quality? It has come to our attention that the photoresponsivity distributes over a wide range with different sheet resistance based on a number of experimental reports. The transport processes of electron-hole pairs induced by photons follow scattering processes in mesoscopic solid-state systems, so the material conductance would be a critical factor in dominating the reported photocurrent response. However, this effect is not yet well studied, and related experimental works are lacking.

To identify the conductance effect on the photocurrent response, we systematically investigated the photocurrent response in topological insulator nanosheets with different conductivity. The photocurrent is linear with light power intensity, and the photocurrent is proportional to the dark current. The higher photocurrent is observed in the nanosheet with higher conductivity. The responsivity is proportional to the nanosheet conductance over two orders. The responsivity is independent of the light power intensity in vacuum, but responsivity drastically decreases at low power intensity in air. The ratio of the responsivity in air to that in vacuum is negatively proportional to the the inverse of the light power intensity. These behaviors are understood as the statistical photocurrent in a system with blocked molecules. The time constant decreases as the thickness increases. This behavior could be understood as the uniformity current flowing process. The charge and discharge time constants of different pressures are determined. A longer time constant is observed in lower atmosphere pressure. The responsivity, *R*, is linear with the nanosheet conductivity. The *R* at *V*=0.1 *V* reaches 731 at nanosheets with higher conductance. These are higher than all reported values in (Sb, Bi)_2_(Te, Se)_3_ topological insulators and low-dimensional materials and only lower than several reported heterostructures.

## Experimental Method

Single crystals of Sb_2_Se_2_Te were grown by a homemade resistance-heated floating zone furnace (RHFZ). The starting raw materials of Sb_2_Se_2_Te were mixed according to the stoichiometric ratio. At first, the stoichiometric mixtures of high-purity elements Sb (99.995%), Se (99.995%), and Te (99.995%) were melted at temperatures of 700 ∼ 800 °C for 20 h and then slowly cooled to room temperature in an evacuated quartz glass tube. The resulting material was used as a feeding rod for the following RHFZ experiment. After growth, the crystals were then furnace cooled to room temperature. The as-grown crystals were cleaved along the basal plane, producing a silvery shining mirror-like surface, and then prepared for further experiments. The Raman, EDS, and XPS spectrum support that the crystal is Sb_2_Se_2_Te. The X-ray diffraction shows sharp peaks that indicate that the Sb_2_Se_2_Te crystal has high crystallinity and uniformity. Our previous works show that physical parameters extracted from ARPES and the quantum SdH oscillation are consistent. These support the Sb_2_Se_2_Te crystal reveals high quality and uniformity.

**Fig. 1:**
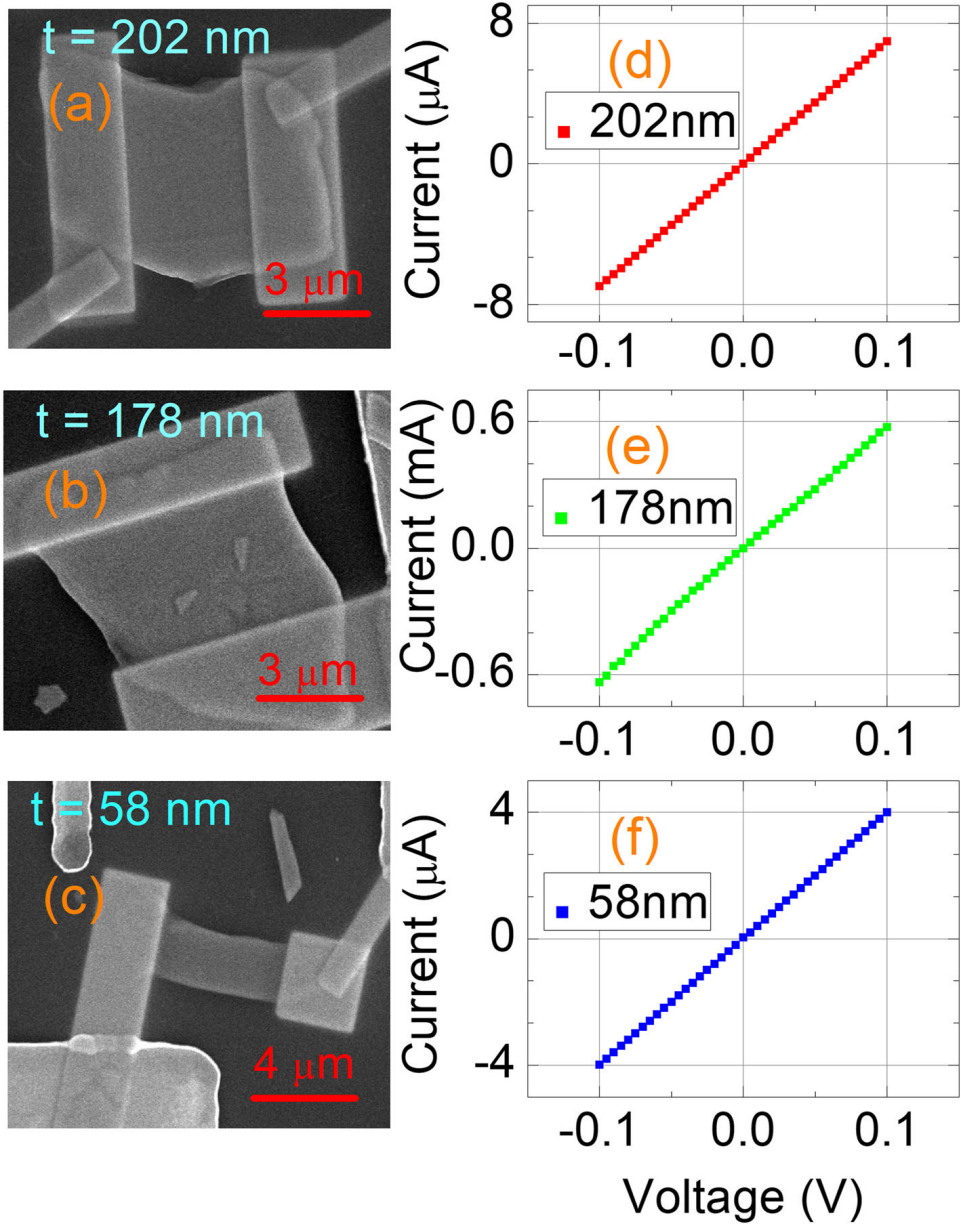
**a**, **b**, and **c** show the SEM pictures of three Sb_2_Se_2_Te nanosheets. The nanosheet thickness is measured by AFM. Two Pt contacts were deposited on a nanosheet to measure the photocurrent. **d**, **e**, and **f** reveal the voltage-current relation, and it is linear. That indicates the ohmic contact between the Pt electrodes and Sb_2_Se_2_Te nanosheets

The Sb_2_Se_2_Te nanosheets were obtained by exfoliating bulk crystals using dicing tape and were then dispersed on the insulating SiO_2_ (300nm)/*n*-Si templates with pre-patterned Ti/Au circuits. Two platinum (Pt) metal contacts were subsequently deposited on the selected Sb_2_Se_2_Te nanosheets using focused-ion beam (FIB) technique. Figure 1a–c shows the SEM pictures of three Sb_2_Se_2_Te nanosheets. The thickness of nanosheets is determined by atomic force microscopy, and measured thickness of three synthesized nanosheets were 58 nm, 178 nm, and 202 nm, respectively. The conductance of these nanosheets were measured by Keithley 4200-SCS. The current were measured as a function of applied voltage in a two-probe method. The I ^+^ and V ^+^ are the same contact point, and the I ^−^ and V ^−^ are the same contact point. To identify the intrinsic conductance effect on the photocurrent response, three nanosheets with different conductance were prepared for the photocurrent measurement.

## Results and Discussion

Figure 1d–f reveals a linear voltage-current relation. This indicates the metallic characteristic and the ohmic contacts between Pt electrodes and nanosheets. The measured conductance, *G*, are 4 × 10^−5^, 0.006, and 7 × 10^−5^ (S) for nanosheets with thicknesses of 202, 178, and 58 nm, respectively. The conductivity is higher than 1000 (S/m) which supports the extremely high crystal quality in our nanosheets.

**Fig. 2:**
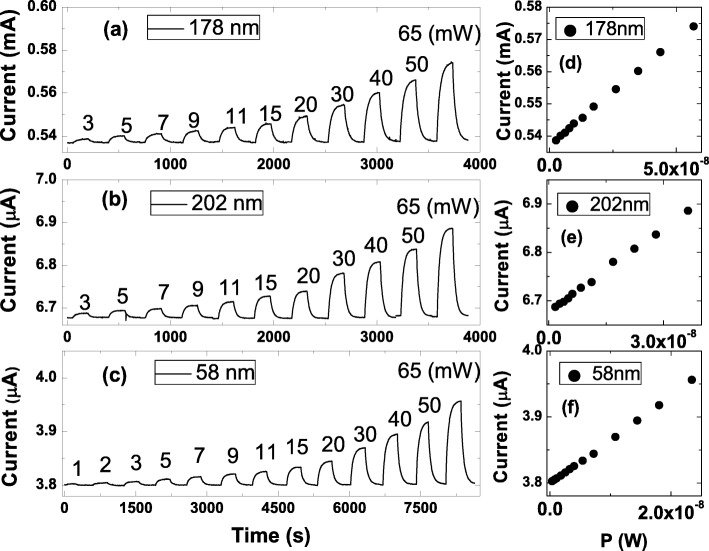
**a**, **b**, and **c** show the measured currents as a function of light power intensity in three samples with different thicknesses. **d**, **e**, and **f** reveal that the measured currents are proportional to the light power intensity. It comes to our attention that the larger *I*_on_ is observed in the nanosheet with larger *I*_off_

Figure 2a–c shows measured currents as a function of the light power intensity. Figure 2d–f reveals that the measured current is proportional to the light power intensity [27, 28]. The relation could be expressed as *I*_on_=*βP*^*α*^+*I*_off_, where the *I*_on_ is the measured currents with light, *I*_off_ is the measured currents without light, *β* is a constant related to the photocurrent response, *P* is the light power intensity, and *α* is a constant related to the light illumination condition between the devices and light. It is worth noting that the larger *I*_on_ is observed in the nanosheet with larger *I*_off_. The photocurrent, *I*_ph_, is defined as *I*_on_−*I*_off_. Table 1 lists the fitting result. It shows that *α*≈1 for all nanosheets with different thicknesses, and that supports the consistent optical characteristics in these nanosheets. It is worth noting that *β*/*G* is 1.1×10^5^±0.2×10^5^ (A /WS) for all nanosheets. This indicates that the observed photocurrent is proportional to the effective conductance. This finding supports that aside from system geometry and the material band structure, the effective conductance of nanosheets would also be a critical factor dominating the photocurrent response.

**Table 1:** Fitting parameters in Sb_2_Se_2_Te nanosheets

Thickness	202 nm	178 nm	58 nm
*β* (A /W)	5.5	641	6.7
*I*_off_ (A)	6.67×10^−6^	5.36×10^−4^	3.80×10^−6^
*β*/*G* (A /WS)	1.37×10^5^	1.06×10^5^	0.96×10^5^
*α*	1	1	1

The *I*_ph_ originates from electron-hole pairs induced by the interaction between injected photons and nanosheets. The induced electrons and holes flow in opposite directions under applied electric bias. The effective *I*_ph_ is proportional to the applied voltage and the amount of electron-hole pairs. More injected photons lead to more electron-hole pairs. The light penetration depth is short and weak depending on the light power intensity. It is reported that the light penetration depth is roughly 20 nm in topological insulators which is smaller than the thickness of our nanosheets [23, 24]. The *I*_ph_ should be independent of the nanosheet thickness when the thickness is larger than the light penetration depth. The nanosheet surface area distributes in a factor of 3, but the observed *I*_ph_ spreads over a two-order difference. Apart from the effective induced electron-hole pairs, the observed different *I*_ph_ should originate from intrinsic properties. In order to exclude extrinsic geometry effects on the *I*_ph_ and quantitatively determine the performance of these nanosheets, the responsivity, *R*, is calculated using the following equation:

1 $$ R = \frac{I_{ph}}{PS},  $$

where *P* and *S* are the light power intensity and the effective area, respectively.

**Fig. 3:**
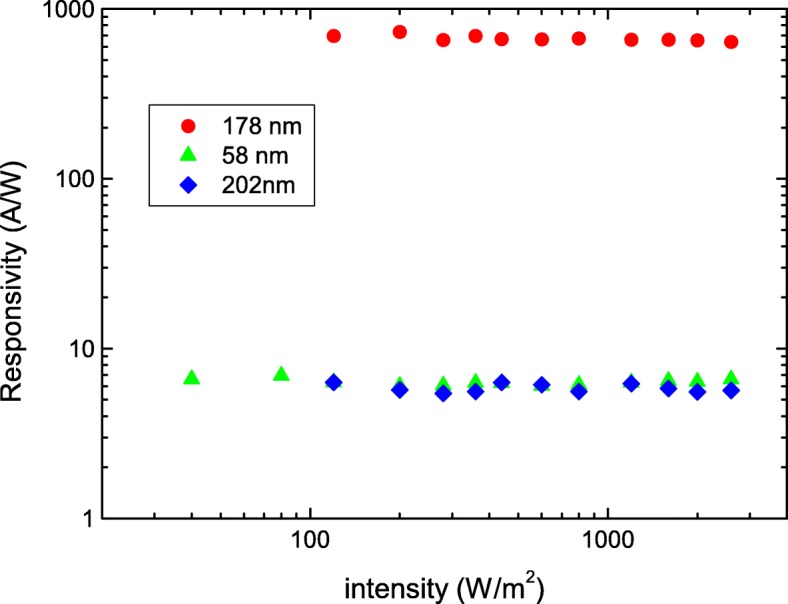
The responsivity of three Sb_2_Se_2_Te nanosheets. It reveals weak dependence of the light power intensity on responsivity. The higher responsivity is observed in the nanosheet with higher conductivity

Figure 3 shows *R* as a function of light power intensity, different from mostly reported that the *R* drastically decreases as light power intensity increases in the Bi-based topological insulators and low-dimensional materials [25, 26]. Our results show that the *R* and *G* are independent of the light power intensity in vacuum. That further supports that the light penetration depth should be shorter than the nanosheet thickness in our experimental conditions. The larger *R* is observed in the nanosheet with higher conductance. This supports that the observed higher photoresponse originates from intrinsic transport characteristics and not from the nanosheet geometry or experimental conditions.

As shown in Fig. 1, the linear voltage-current relation supports that nanosheets reveal a metallic behavior. The light-induced electron-hole pairs would travel to two electrode contacts due to the applied voltage bias [27–29]. Following Ohm’s law, the related photocurrent could be determined through the relation *I*_ph_=*VG* where *V* is the applied voltage bias between two electrodes. The *I*_ph_ is proportional to the *G*.

**Fig. 4:**
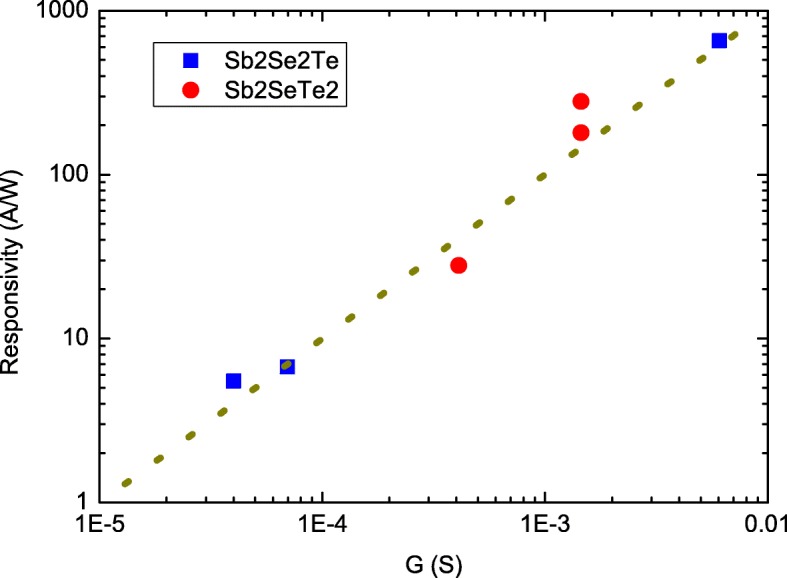
Responsivity as a function of nanosheet conductance. It shows responsivity is proportional to the nanosheet conductance. The Sb_2_SeTe_2_ data are from our reported values

Figure 4 reveals the *R* as a function of the *G* in a log-log plot. The data points of Sb_2_Se_2_Te are the measured results in this work, and data points of Sb_2_SeTe_2_ are extracted from our previous work under the same crystal growth conditions and measurement setups [27]. The thickness of Sb_2_SeTe_2_ nanosheets are about 180 nm. The wavelength is 532 nm. Both Sb_2_Se_2_Te and Sb_2_SeTe_2_ show that *R* is independent of the light power intensity. These data points follow the tendency of the dot line over a wide range of the nanosheet conductance. This supports that *R* is proportional to the *G*, which is consistent with our proposal.

A system with higher photocurrent response is greatly preferred for potential applications. As well as looking for new materials or systems with specific band structure and band gap, a proper treatment on a system would be also suitable methods to enhance photoresponse. Our experimental results support that the intrinsic electric conductance would be a critical factor to optimize the photocurrent response. This might be achieved through appropriate growth conditions. As shown in Fig. 4, the photocurrent is 2 orders enhanced through conductance adjustment. This study could guide other researchers into constructing a suitable guideline in selecting a better system for further experimental studies through a simple electric test.

The *R* and detectivity at *V*=0.1*V* reaches 731 and 2.6×10^10^ at the nanosheet with higher conductivity. These photoresponses are larger than all reported values in (Sb, Bi)_2_(Te, Se)_3_ topological insulators and low-dimensional materials [27, 28] and only lower than several reported heterostructures. Recently, low-dimensional materials caught a great attention in the field of photocurrent. It comes to our attention that the reported conductivity in these low-dimensional materials are extremely high. This is consistent with our experimental result that the conductivity would be a critical factor dominating the photocurrent response.

**Fig. 5:**
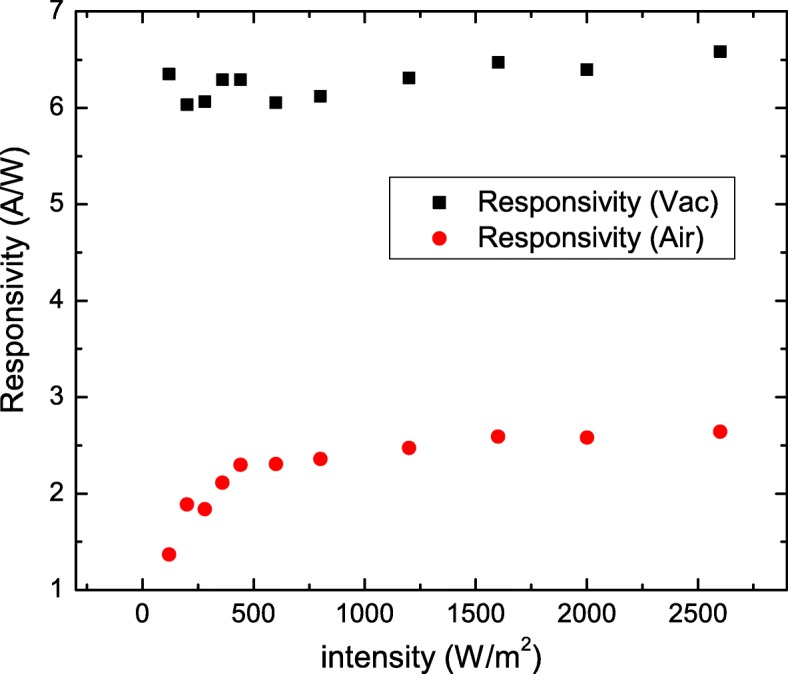
Responsivity and photoconductive gain as functions of the light power intensity at a wavelength of 532 nm. The responsivity is weak light power intensity dependence in vacuum. The responsivity decreases as the light power intensity decreases in atmosphere when the light power intensity is lower than 500 *W*/*m*^2^

Figure 5 depicts *R* as functions of the light power intensity in vacuum and atmosphere. It comes to our attention the *R* drastically decreases when the light intensity is lower than 500 Wm ^−2^ in atmosphere. This supports that this decreasing *R* at low light intensity in atmosphere might come from the influence of the adsorbed molecular on the surface of our Sb_2_Se_2_Te nanosheet.

The photoresponse would be extremely sensitive to the condition of sample surface. In addition to the reduction of the effective response area, surface defects and oxidation might reduce carrier mobility and lifetime.

Recently, it is reported that adsorbed molecules on the surface of Bi_2_Se_3_ topological insulators bend the structure and lead to an additional 2DEG. This induced 2DEG would enhance the effective carrier mobility [30]. A system with higher carrier mobility might decrease the carrier transit time and produce a higher photocurrent. On the other hand, the enhanced carrier mobility should be independent of the light power intensity. These support that the observed *R* suppression is mainly from the effective shining area, not from the intrinsic transport characteristics. Thus, it is believed that the drop of *R* in air at low light power intensity should be more related to adsorbed molecular shadow effect than the intrinsic complex carrier relaxation effect.

**Fig. 6:**
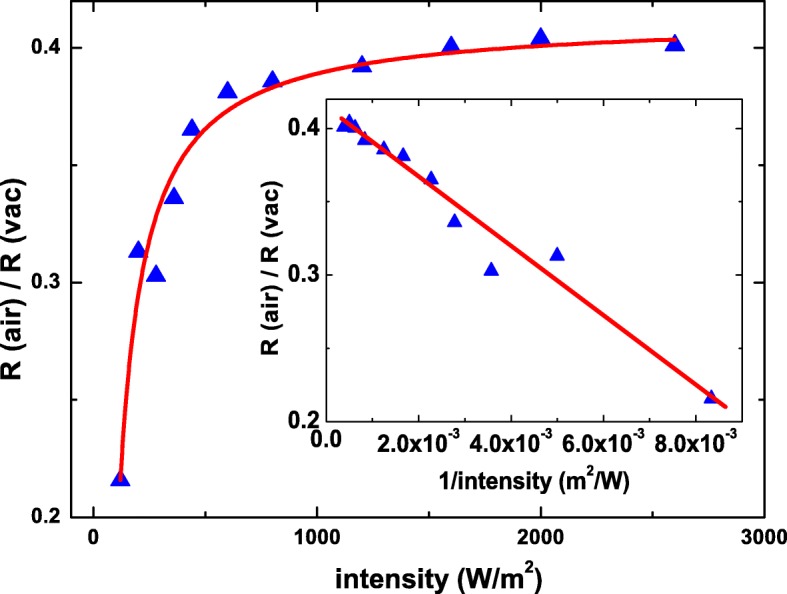
The ratio of the responsivity in air to that in vacuum as a function of the light power intensity. The data points go well with the theoretical predication. The inset shows the ratio of the responsivity in air to that in vacuum is negatively proportional to the the inverse of the light power intensity

The definition of the responsivity is the ratio of the induced carrier to the incident photons, and it also could be expressed as $R=\eta \frac {q}{hf},$ where *q*, *hf*, and *η* are the carrier charge, the photo energy, and the quantum efficiency, respectively. The *η* is directly related to the material properties and the light wavelength. To exclude other extrinsic and intrinsic effects and optimize the molecule shadow effect, the *R*(*air*)/*R*(*vac*) is plot as a function of the light power intensity. As shown in Fig. 6, the ratio increases as power intensity increases and gradually saturates at high power intensity.

We propose a model that the incident photon number is *Y*, the *m* photons interact with material, and *n* photons are blockaded by adsorbed molecular on the surface. That is, *Y*=*m*+*n*. The *Z* is the average induced photocurrent carrier number by one photon. In the extremely weak light intensity, the photo number is much less than the total molecular unit, the effective photocurrent should follow the statistical calculation, and the result supports that the quantum efficiency, *η*, could be expressed as

2 $$ \eta(air)=\left(1-\frac{n}{2Y}\right)Z.  $$

This statically calculation supports that the effective photocurrent would be strongly related to the light power intensity in the limit of the weak light power intensity and long relaxation time; the photon number might be smaller than the “photo carrier creator.” The effective photocurrent could be expressed as

3 $$ \frac{R(air)}{R(vac)} \propto \left(1-\frac{n}{2Y}\right)  $$

The *Y* is directly proportional to the light power intensity. The *R*(*air*)/*R*(*vac*) is negatively proportional to the inverse of *Y* and is weakly dependent on the *Y* in the situation of the *Y*≫*n*. As shown in Fig. 6, it clearly reveals that the measured data points go well with the theoretical equation, and the inset shows that the data points are negatively proportional to the inverse of the light power intensity. These support that the observed photocurrent drop mainly comes from the shadow effect of adsorbed molecules on the surface. The $\frac {R(air)}{R(vac)}$ is roughly 0.4 at high power intensity and that indicates the surface covers with adsorbed molecules by 40%.

**Fig. 7:**
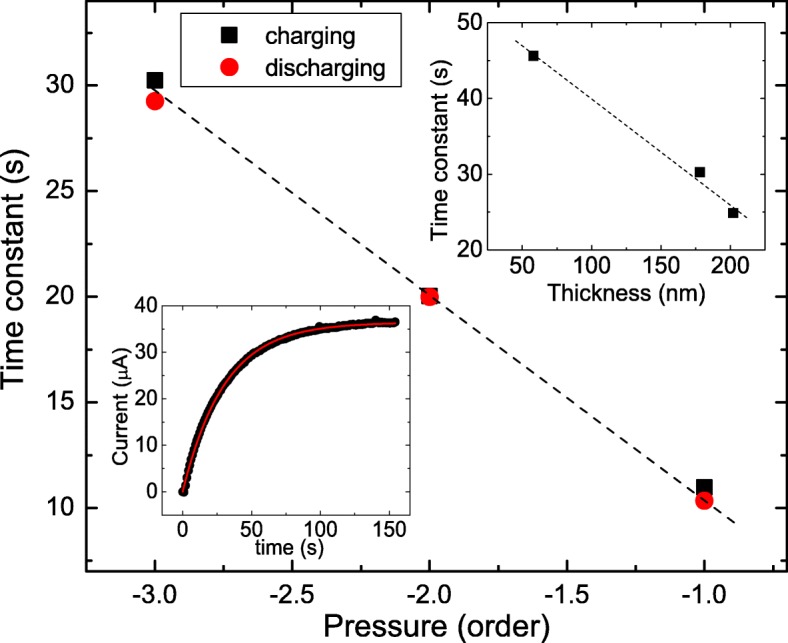
The bottom-left inset shows the photocurrent as a function of time in the charging process, and it goes well with the fitting line. The top-right inset shows the charge time constant as a function of thickness. The charge and discharge time constant as a function of pressure

The bottom-left inset of Fig. 7 shows the photocurrent as a function of time. The charging process could be described by the *e*^−*t*/*k*^, where *k* is characteristic time constant. Our experimental result reveals that the measured photocurrent goes well with the fitting line. The top-right inset shows the extracted charge time constant as a function of thickness. It reveals that the time constant decreases as the thickness increases. This behavior could be understood as the uniformity current flowing process [27, 28]. On the other hand, the charge and discharge time constants of different atmosphere pressures are determined. It shows that charge time constant is roughly the same as the discharge time constants, and longer time constant is observed in lower atmosphere pressure.

## Conclusion

The photocurrent was performed in the Sb_2_Se_2_Te topological insulator with different conductance at a wavelength of 532 nm. The photocurrent is linear with light power intensity, and the photocurrent is proportional to the dark current. Higher photocurrent is observed in the nanosheet with higher conductance. The responsivity is proportional to the nanosheet conductivity. The responsivity is independent of the light power intensity in vacuum, but responsivity drastically decreases at low power intensity in air, that is, in contrast to most reported results. The ratio of the responsivity in air to that in vacuum is negatively proportional to the the inverse of the light power intensity. These behaviors are understood as the statistical photocurrent in a system with blocked molecules. Following the theoretical model, the surface covers with adsorbed molecules by 40% in air. The time constant decreases as the thickness increases. This behavior could be understood as the uniformity current flowing process. The charge and discharge time constants of different pressures are determined. A longer time constant is observed in lower atmosphere pressure. The *R* and detectivity at *V*=0.1*V* reaches 731 and 2.6×10^10^ at the nanosheet with higher conductivity. These are higher than all reported values in (Sb, Bi)_2_(Te, Se)_3_ topological insulators and low-dimensional materials and only lower than several reported heterostructures.

## Abbreviations

ARPES: Angle resolved photoemission spectroscopy
EDS: Energy-dispersive X-ray spectroscopy
SdH: Shubnikov-de Haas
XPS: X-ray photoelectron spectroscopy
